# MIND Diet Impact on Multiple Sclerosis Patients: Biochemical Changes after Nutritional Intervention

**DOI:** 10.3390/ijms251810009

**Published:** 2024-09-17

**Authors:** Ainoa Navarrete-Pérez, Sara Gómez-Melero, Begoña Mª Escribano, Alejandro Galvao-Carmona, Cristina Conde-Gavilán, Mª Ángeles Peña-Toledo, Noelia Villarrubia, Luisa Mª Villar, Isaac Túnez, Eduardo Agüera-Morales, Javier Caballero-Villarraso

**Affiliations:** 1 Maimónides Biomedical Research Institute of Córdoba (IMIBIC), 14004 Córdoba, Spain; ep2napea@uco.es (A.N.-P.); sara.gomez.melero@gmail.com (S.G.-M.); am1esdub@uco.es (B.M.E.); agalvao@uloyola.es (A.G.-C.); cristinaconde84@gmail.com (C.C.-G.); angelespe87@gmail.com (M.Á.P.-T.); itunez@uco.es (I.T.); 2 Department of Cell Biology, Physiology and Immunology, Faculty of Veterinary Medicine, Universidad of Córdoba, 14071 Córdoba, Spain; 3 Department of Psychology, Universidad Loyola Andalucía, 41704 Sevilla, Spain; 4 Immunology Department, Hospital Universitario Ramón y Cajal, Red Española de Esclerosis Múltiple, Red de Enfermedades Inflamatorias, Instituto de Salud Carlos III, IRYCIS, 28034 Madrid, Spain; noelia.villarrubia@salud.madrid.org (N.V.); luisamaria.villar@salud.madrid.org (L.M.V.); 5 Department of Biochemistry and Molecular Biology, Universidad of Córdoba, 14071 Córdoba, Spain; 6 Neurology Service, Reina Sofia University Hospital, 14004 Córdoba, Spain; 7 Clinical Analyses Service, Reina Sofía University Hospital, 14004 Córdoba, Spain

**Keywords:** MIND diet, multiple sclerosis, dietary intervention, lifestyles, quality of life, fatigue, serum biomarkers, neurotrophic factors, neurofilaments, oxidative stress

## Abstract

There is substantial evidence supporting the neuroprotective effects of the MIND diet in neurodegenerative diseases like Parkinson’s and Alzheimer’s. Our aim was to evaluate the impact of a nutritional intervention (NI) with this diet on multiple sclerosis (MS) patients. The study was conducted in two stages. In the first stage, two groups were included: MS patients before the NI (group A) and healthy control subjects (group B). In this stage, groups (A) and (B) were compared (case–control study). In the second stage, group (A) was assessed after the NI, with comparisons made between baseline and final measurements (before-and-after study). In the case–control stage (baseline evaluation), we found significant differences in fatigue scores (*p* < 0.001), adherence to the MIND diet (*p* < 0.001), the serum levels of brain-derived neurotrophic factor (BDNF) (*p* < 0.001), and higher oxidative status in the MS group, with lower levels of reduced glutathione (*p* < 0.001), reduced/oxidised glutathione ratio (*p* < 0.001), and elevated levels of lipoperoxidation (*p* < 0.002) and 8-hydroxy-2′-deoxyguanosine (*p* < 0.025). The before-and-after intervention stage showed improvements in fatigue scores (*p* < 0.001) and physical quality-of-life scores (MSQOL-54) (*p* < 0.022), along with decreases in the serum levels of glial-derived neurotrophic factor (GDNF) (*p* < 0.041), lipoperoxidation (*p* < 0.046), and 8-hydroxy-2′-deoxyguanosine (*p* < 0.05). Consumption of the MIND diet is linked to clinical and biochemical improvement in MS patients.

## 1. Introduction

Multiple sclerosis (MS) is the most common demyelinating disease of the central nervous system (CNS) [1]. It is autoimmune in nature, although the precise mechanisms underlying its aetiopathogenesis are not fully understood. Among these mechanisms, several theories have been postulated, with the most widely accepted being the peripheral activation of auto-reactive T cells by various aetiological agents, leading to neuronal demyelination and promoting an inflammatory environment. This is accompanied by a pro-oxidant and pro-inflammatory environment despite the production of antioxidants [2]. The interaction between neurodegeneration and oxidative stress, which promotes inflammation, neurodegeneration, and demyelination, has been described. As a result of demyelination, nerve impulse conduction is slowed, manifesting as sensory and motor symptoms, which may also be accompanied by cognitive impairment, such as memory loss, and depressive symptoms [3].

The ability of immune cells to release various neurotrophic factors has been proposed as a neuroprotective mechanism. Some of these neurotrophic factors include brain-derived neurotrophic factor (BDNF), neuronal growth factor (NGF), and glial-derived neurotrophic factor (GDNF) [4].

Previous studies have linked BDNF to lower serum levels in people with MS compared with healthy individuals [5]. Higher serum levels of BDNF have also been observed during disease flare-ups and in the recovery phase of the disease, compared with the levels observed in stable phases [4]. These findings support the hypothesis that higher BDNF levels are associated with active inflammation, although many other authors have found no direct relationship between serum BDNF levels and the relapsing and remitting phases of the disease [6].

On the other hand, GDNF has been postulated to be a neuroprotective factor, with higher levels being observed in Alzheimer’s disease, possibly as an adaptive mechanism to compensate for neuronal damage. Additionally, some authors have observed higher levels of GDNF in individuals with cognitive impairment compared with those without [7]. In recent years, other serum biomarkers have been used to provide insights into MS risk and progression, such as glial fibrillary acidic protein (GFAP), whose levels increase in people with inflammatory neuronal damage due to possible new flare-ups and decrease when treatments keep the disease stable [7].

Proteins that are a structural part of neurons, such as neurofilaments (Nfl) have been proposed as biomarkers of axonal damage. In individuals with MS, an increase in Nfl blood levels has been observed just after a flare-up, which has been correlated with the degree of subsequent disability. In addition, the type of treatment appears to influence Nfl levels, with monoclonal antibody therapies showing greater effectiveness in reducing NfL levels. However, more recent studies agree that Nfl levels do not fall to normal levels with these therapies and a small steady increase may be observed at baseline, which may reflect disease progression itself [8].

To date, there is no curative treatment for MS and the available therapeutic options only slow disease progression. For this reason, the use of antioxidants through supplementation or diet could be a therapeutic resource to antagonise these effects [9].

Diet shows promise in regulating the course of the disease. By comparing different intake patterns, it has been observed that some Western diets associated with a sedentary lifestyle may promote a pro-inflammatory and pro-oxidant environment. In contrast, the Mediterranean diet, together with regular physical exercise, may have an anti-inflammatory effect and promote the activation of antioxidant metabolic mechanisms [10].

In addition, certain dietary modifications, with an increased intake of plant-based foods or following nutritional patterns such as the Mediterranean Diet, have been shown to reduce MS flare-up rate, disability according to the EDSS (‘Expanded Disability Status Scale’), and fatigue according to the MFIS (‘Modified Fatigue Impact Scale’) and improve quality of life according to the MSQOL-54 (‘Multiple Sclerosis Quality Of Life 54’) scale [11,12].

Combining the antioxidant and anti-inflammatory features of the Mediterranean Diet and the DASH (‘Dietary Approaches to Stop Hypertension’) diet, a new dietary model has emerged, known as the MIND (‘Mediterranean-DASH Intervention for Neurodegenerative Delay’) diet, described in 2015 by Morris et al. [13]. This could have a positive impact on the clinical course of MS, as it has shown beneficial effects in other neurodegenerative diseases. In this regard, it has been shown to reduce the risk of Alzheimer’s disease or delay the age of onset in Parkinson’s disease [14], as well as improving cognitive function (something very commonly affected in people with MS) [15,16,17] or reducing depressive symptoms in the general population [18,19].

This diet is characterised by an increased intake of berries and green leafy vegetables, which are the foods linked to greater neuroprotection because they contain certain components with important antioxidant action due to their important antioxidant properties [16]. In addition, some of the micronutrients present in this diet, such as vitamin E and zexanthine-lutein, are integral components of biological membranes that regulate the levels of reactive oxygen species (ROS) and maintain redox homeostasis [20]. The diet also shares similarities with the Mediterranean Diet, including a high content of omega-3 fatty acids (associated with better cognitive function) and extra virgin olive oil (EVOO), both of which have been associated with lower prevalence of Alzheimer’s disease [21].

The aim of the present study is to evaluate the impact of a nutritional intervention with the MIND diet in patients with MS at the biochemical and clinical levels.

## 2. Results

### 2.1. Characteristics of Participants

The average duration of disease progression was 8.52 ± 5.74 years overall, with an average of 8.23 ± 5.84 years for women and 9.14 ± 5.68 years for men. Among the participants, 38.63% were on Ocrelizumab, 13.63% on Alemtuzumab, 31.81% on Natalizumab, 2.27% on Rituximab, and 13.63% on Cladribine. None was under interferon treatment.

**Figure 1:**
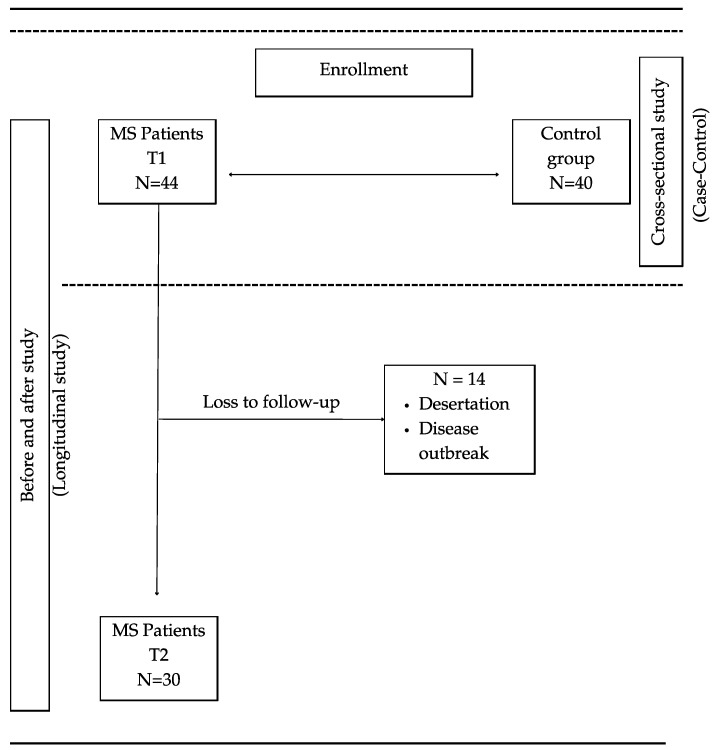
Study design.

### 2.2. Case–Control Study

#### 2.2.1. Lifestyles and Scale Scores

**Figure 2:**
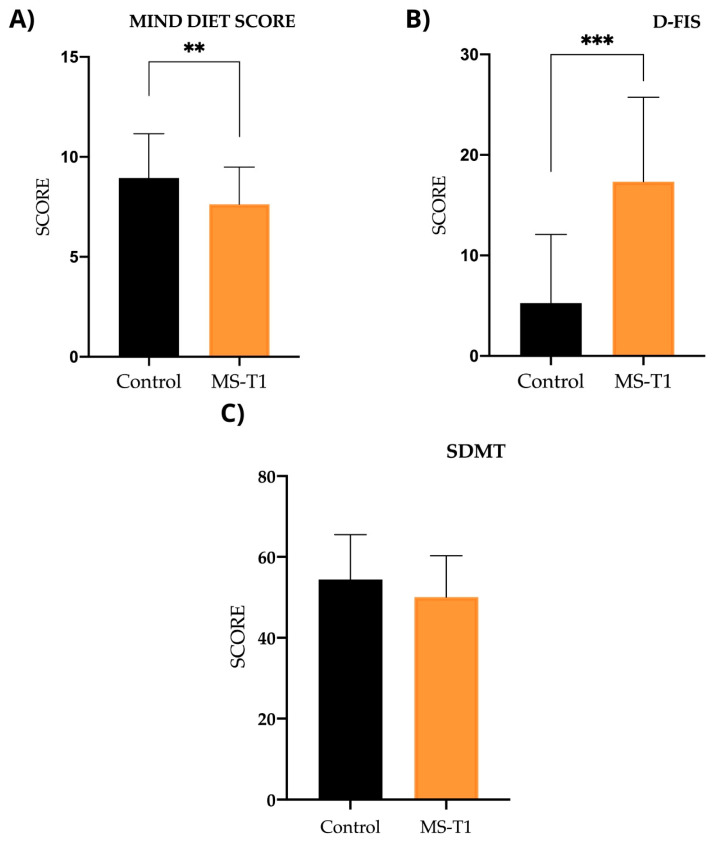
Scores for adherence to MIND diet, D-FIS scale, and SDMT. (**A**) MIND (Mediterranean-DASH Intervention for Neurodegenerative Delay): comparison between controls and MS patients prior to nutritional intervention (MS-T1) (*p* < 0.01). (**B**) D-FIS (Fatigue Impact Scale for Daily Use): comparison between controls and MS patients before nutritional intervention (*p* < 0.001). (**C**) SDMT (Symbol Digit Modalities Test): comparison between controls and patients with MS prior to nutritional intervention (*p* = 0.074). ** *p*< 0.01; *** *p*< 0.001.

In terms of habits and socio-demographic characteristics in the case–control study, differences were observed between the two groups in work status as well as in dietary habits, with the control group presenting an eating pattern more similar to the MIND diet (Figure 2), with scores of 9.07 ± 2.09 for the control group and 7.62 ± 1.86 for the MS group (*p* < 0.001). For fatigue, there were significant differences (*p* < 0.001), with lower scores in the control group (5.25 ± 6.83) compared with the MS group (17.34 ± 8.40); however, for the Symbol Digit Modalities Test (SDMT), there were no differences between the control group (54.39 ± 11.14) and the MS group (50.05 ± 10.25) (*p* < 0.074). In addition, there were significant differences in smoking habits, which were less frequent in the control group (Table 1).

#### 2.2.2. Measurements of Serum Biochemical Magnitudes

In the case–control study, we observed significant differences in albumin, high-density lipoproteins (HDLs), low-density lipoproteins (LDLs), creatine kinase (CK), chlorine (Cl), calcium (Ca), apolipoprotein A-I (Apo A1), C-reactive protein (CRP), and insulin. As for CRP between the control group and the MS group, the levels were lower in the control group, as was the case for LDLs (Table 2).

#### 2.2.3. Neurofilaments and Neurotrophic Factors

Significant differences were observed in the BDNF levels, which were much higher in the MS group (Table 3).

#### 2.2.4. Oxidative Stress Parameters

**Figure 3:**
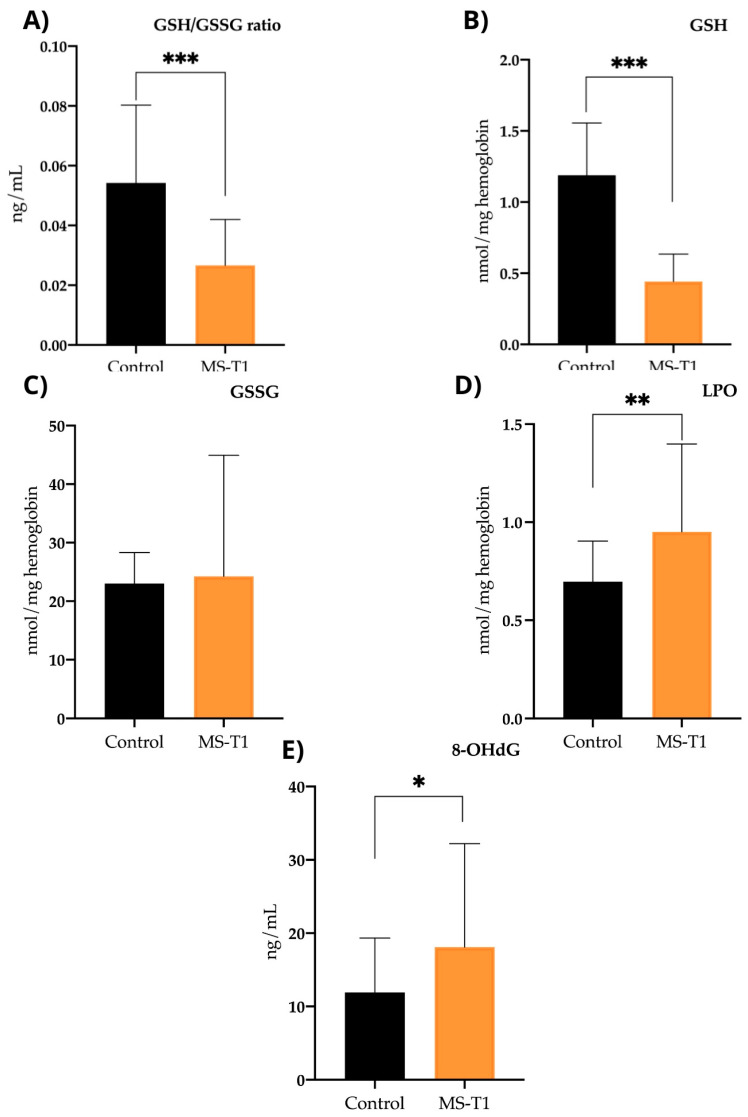
Case–control study: oxidative stress biomarkers. (**A**) GSH/GSSG ratio (reduced glutathione/oxidized glutathione ratio): serum levels in control group and in MS patients before nutritional intervention (*p* < 0.001). (**B**) GSH (reduced glutathione): serum levels in control group and MS patients before nutritional intervention (*p* < 0.001). (**C**) GSSG (oxidized glutathione): serum levels in control group and MS patients prior to nutritional intervention (*p* < 0.723). (**D**) LPOs (lipoperoxidation products): serum levels in control group and MS patients before nutritional intervention (*p* < 0.002). (**E**) 8-OHdG (8-hydroxy-2′-deoxyguanosine): serum levels in control group and MS patients before nutritional intervention. * p< 0.05; ** *p*< 0.01; *** *p*< 0.001.

In the control group, we observed significant differences compared with the MS group in reduced glutathione (GSH), with mean values of 0.44 ± 0.19 for the MS group and 1.18 ± 0.36 for the control group (*p* < 0.001). We also observed differences in the reduced glutathione/oxidised glutathione (GSH/GSSG) ratio, with the MS group showing a ratio of 0.026 ± 0.015 and the control a ratio of 0.054 ± 0.026 (*p* < 0.001) (Figure 3).

As for lipoperoxidation product (LPO) levels, we observed statistically significant differences (*p* < 0.002), with higher values in the MS group (0.95 ± 0.44) than in the control group (0.69 ± 0.21). On the other hand, regarding 8-OHdG levels, we observed a more oxidative profile in the MS group (18.1 ± 14.12) and less antioxidants than in the control group (12.29 ± 7.67), showing significant differences (*p* < 0.025). However, regarding the levels of oxidised glutathione (GSSG), we observed no differences (*p* < 0.723), with similar levels between the MS group (24.23 ± 20.71) and the control group (23.02 ± 5.27) (Figure 3).

### 2.3. Follow-Up Study: Assessment after Nutritional Intervention

#### 2.3.1. Lifestyles and Scale Scores

In terms of lifestyle habits, a slight improvement in nighttime sleep was observed after the nutritional intervention, with an average increase of 2.26% in the number of hours of sleep. In terms of physical activity, participants maintained the frequency and intensity of physical exercise (Table 4).

**Figure 4:**
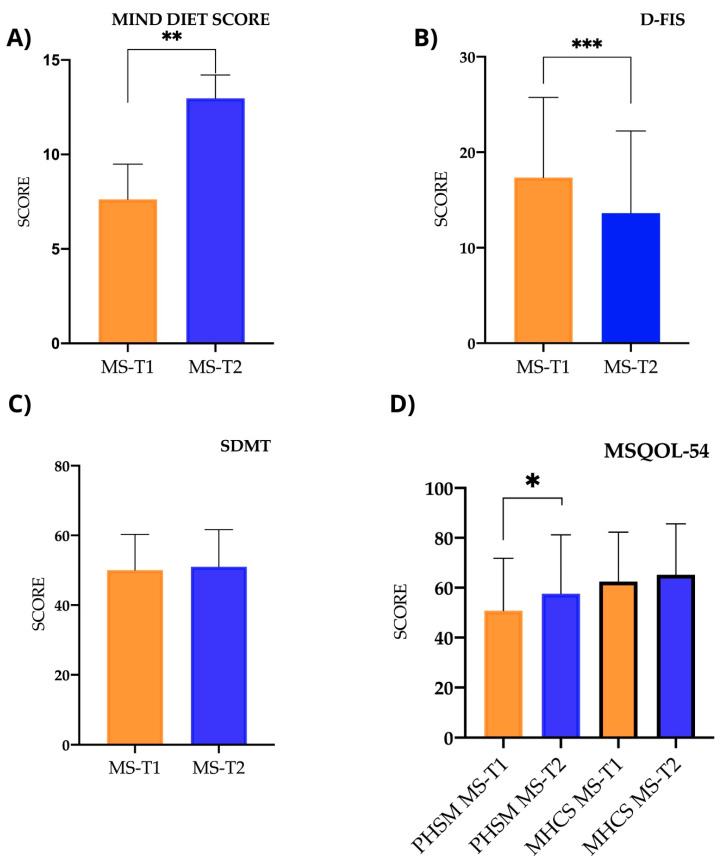
Scores for dietary adherence to MIND diet, SDMT, and D-FIS and MSQOL-54 scales. (**A**) MIND (Mediterranean-DASH Intervention for Neurodegenerative Delay): scores in MS patients at time 1 and time 2 (*p* < 0.001). (**B**) D-FIS (Fatigue Impact Scale for Daily Use): scores in MS patients at time 1 and time 2 (*p* < 0.001). (**C**) SDMT (Symbol Digit Modalities Test): scores in MS patients at time 1 and time 2 (*p* < 0.072). (**D**) MQOL-54 (Multiple Sclerosis Quality of Life-54): MS patient scores at time 1 and time 2 of Physical Health Composite Score (PHSM) (*p* < 0.022) and Mental Health Composite Score (MHCS) (*p* < 0.419). * p< 0.05; ** *p*< 0.01; *** *p*< 0.001.

After the nutritional intervention, adherence to the MIND diet improved significantly, increasing from low (7.61 ± 1.88) to good (12.98 ± 1.22) (*p* < 0.001). There was also a significant decrease in fatigue (*p* < 0.001), with scores changing from 17.14 ± 8.48 before the nutritional intervention to 13.89 ± 8.56 afterwards. However, as in the case–control study, the score for the SDMT showed little change (pre-intervention, 49.67 ± 10.44, and post-intervention, 51 ± 10.65; *p* < 0.072). Regarding the MSQOL-54 quality-of-life assessment, there was a significant improvement (*p* < 0.022) in the physical subtotal score, which increased from 51.72 ± 20.44 before the intervention to 57.62 ± 23.59 afterwards. However, the mental subtotal score did not show a significant change (pre-intervention, 62.88 ± 20.18, and post-intervention, 65.24 ± 20.40; *p* < 0.419) (Figure 4).

#### 2.3.2. Measurements of Serum Biochemical Magnitudes

After the nutritional intervention, participants experienced a significant decrease in LDL cholesterol and triglyceride levels. A decrease in CRP was also observed, although it was not significant, as was the case for other parameters, such as Apo A-I, total cholesterol, and insulin (Table 5).

#### 2.3.3. Neurofilaments and Neurotrophic Factors

In the follow-up study, we only observed a difference in GDNF, which showed a statistically significant decrease in MS patients after the nutritional intervention (*p* < 0.041) (Table 6).

**Figure 5:**
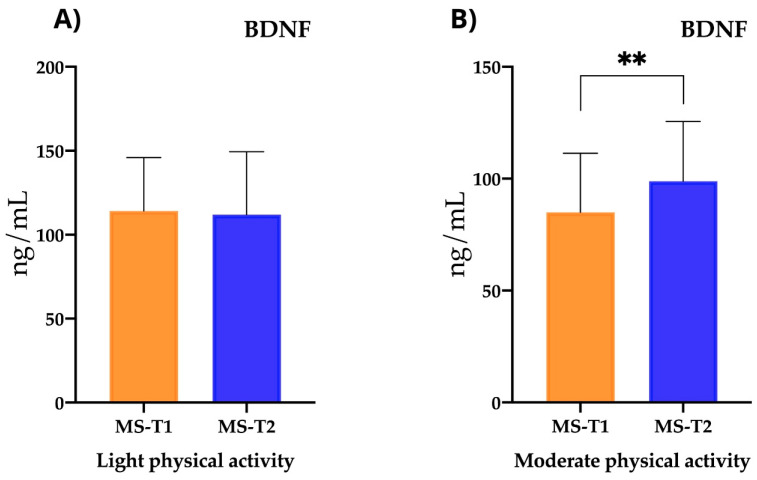
BDNF levels in the follow-up study as a function of physical activity. (**A**) BDNF (brain-derived neurotrophic factor) for light physical activity (*p* < 0.706). (**B**) BDNF (brain-derived neurotrophic factor) for moderate physical activity (*p* < 0.008). ** *p*< 0.01.

However, for those subjects performing moderate–intense physical activity, before the nutritional intervention, the BDNF levels were 80.28 ± 26.62 ng/mL, and after the intervention, they increased significantly to 93.34 ± 18.62 ng/mL (*p* < 0.008). In contrast, those who engaged in light physical activity had pre-existing BDNF levels of 124.5 ± 27.36 ng/mL, and after the nutritional intervention, these levels decreased to 121 ± 34.85 ng/mL (*p* < 0.706) (Figure 5).

#### 2.3.4. Oxidative Stress Parameters

**Figure 6:**
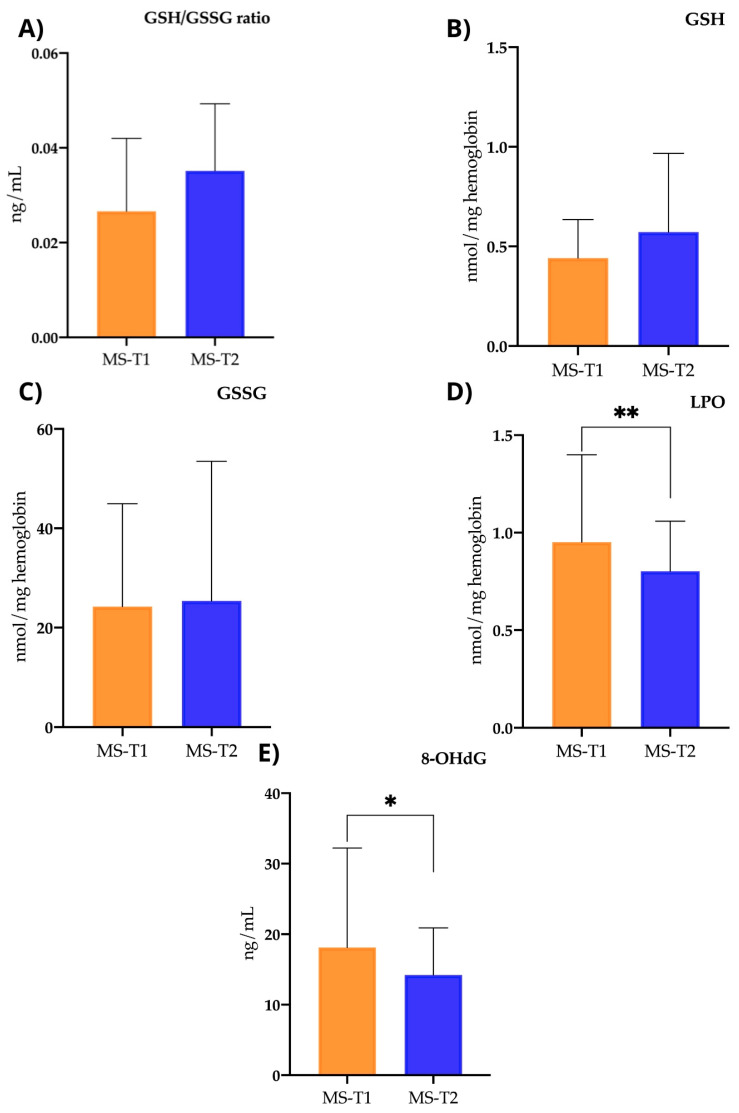
Before-and-after study: oxidative stress biomarkers. (**A**) GSH/GSSG ratio (oxidized glutathione/reduced glutathione ratio): serum levels in MS patients at time 1 (pre-intervention) and time 2 (post-intervention) (*p* < 0.281). (**B**) GSH (reduced glutathione): serum levels in MS patients at time 1 (pre-intervention) and time 2 (post-intervention) (*p* < 0.065). (**C**) GSSG (oxidized glutathione): serum levels in MS patients at time 1 (pre-intervention) and time 2 (post-intervention) (*p* < 0.750). (**D**) LPOs (lipoperoxidation products): serum levels in MS patients at time 1 (pre-intervention) and time 2 (post-intervention) (*p* < 0.046). (**E**) 8-OHdG (8-hydroxy-2′-deoxyguanosine): serum levels in MS patients at time 1 (pre-intervention) and time 2 (post-intervention) (*p* < 0.05). * p< 0.05; ** *p*< 0.01.

After the nutritional intervention, there was a statistically significant decrease in biomarkers of oxidative stress such as LPOs and 8-OHdG. In the case of LPOs, the levels before and after the intervention were 0.97 ± 0.5 and 0.8 ± 0.25, respectively, showing a significant decrease (*p* < 0.046). In the case of 8-OHdG, the values went from 18.48 ± 14.78 to 14.21 ± 6.69 after the nutritional intervention, also showing a significant decrease (*p* < 0.05) (Figure 6).

No significant changes were observed in parameters related to antioxidant status, such as reduced glutathione (GSH), which had pre-intervention values of 0.44 ± 0.19 and increased to 0.57 ± 0.39 (*p* < 0.065). There were also no significant differences in oxidised glutathione (GSSC) (pre-intervention, 24.14 ± 25.03, and post-intervention, 25.37 ± 28.13) (*p* < 0.750) nor in the GSH/GSSG ratio (pre-intervention, 0.031 ± 0.015, and post-intervention, 0.035 ± 0.014) (*p* < 0.281) (Figure 6).

## 3. Discussion

In the present study, it can be observed that a nutritional intervention with the MIND diet in MS patients is associated with changes at both the biochemical (in terms of serum biomarkers) and clinical (in terms of symptoms) levels. Regarding clinical changes, the MIND dietary pattern appears to reduce fatigue, a very common symptom in this disease. Adherence to the MIND diet seems to improve physical aspects related to quality of life and fatigue, although in our case, unlike in the study in MS patients by Mousavi-Shirazi-Farz Z. et al., we did not find a significant improvement in mental quality-of-life scales, although we did find an improvement in physical and fatigue scales, as already described by these authors [11]. In other previous studies with nutritional interventions in MS, the SDMT scale score increased 12 weeks after the start of the intervention, but it was not until week 24 that significant differences were observed [22].

The results obtained by these research groups may suggest that in our study, the SDMT score increased after the dietary intervention, but this increase was not significant, possibly because the follow-up period did not extend to 24 weeks.

It should be noted that this is the first study in the biomedical literature where a nutritional intervention with the MIND diet (with intensive personalised coaching) is performed in MS patients, as previous research at most assessed the degree of adherence to a dietary pattern (Mediterranean Diet or anti-inflammatory diet), as these were mainly observational studies [23]. Some authors performed nutritional interventions but with other types of semi-vegetarian diets, the Paleo Diet, with dietary supplements, or with the Swank diet [10,11,22].

Some unhealthy lifestyle habits, such as smoking, have been associated with a higher degree of disability according to the EDSS scale. In our study, we observed that this habit is more present in the MS group than in the control group, as described in other studies [24]. This may be due to the fact that the healthy population has fewer unhealthy habits related to risk factors for various diseases (including MS). If the selection of people in the control group maintains the representativeness of the general population, this may be one reason why the MS group tends to have more smokers. In our hospital, MS patients are encouraged to give up smoking (but unfortunately often do not). In the present study, the subjects in the MS group did not quit smoking and did not reduce cigarette consumption during the intervention period (NI).

Regarding the biochemical profile, when comparing the MS group with the control group, we observed higher albumin levels in the MS group. After the nutritional intervention, these values decreased and became closer to those of the control group. This finding is somewhat surprising, as some authors have previously reported that blood albumin levels are generally lower in people with MS compared with healthy individuals, attributing this to inflammation and disruption of the blood–brain barrier [25]. However, other authors suggest that increased albumin levels at disease onset are associated with increased brain atrophy and disability following the onset of MS [26]. It has also been suggested that elevated serum albumin levels may be associated with a lower risk of MS onset [27].

In our study, after 12 weeks of nutritional intervention with the MIND diet, an improvement in the lipid profile was observed, as was also described in the healthy population in other studies, where LDL, HDL, triglyceride (TG), and total cholesterol levels were measured together with the frequency of food consumption after the participants followed a nutritional pattern similar to the MIND diet [28]. Additionally, in the control group, HDL-cholesterol values were higher, and LDL levels were lower than in the MS group. After the nutritional intervention, the TG and LDL levels in the MS group decreased significantly, even below the baseline values of the control group; these parameters are typically associated with metabolic syndrome and increased cardiovascular risk. This overall improvement in the lipid profile could perhaps help to improve the SDMT score, which we observed to have increased after the intervention (although not significantly). Other authors, such as Noodi H. et al., have been able to confirm this association between the lipid profile and the SDMT in people with MS [29]. Regarding baseline apolipoprotein A-I levels, differences were observed between the MS group and the control group, with higher levels being observed in the control group. This finding supports the metabolic alterations observed in previous studies, where there appeared to be certain degree of insulin resistance in MS patients [30]. The same is true for the CRP levels, which were higher in the MS group. This has been observed in various studies and may be related to the underlying inflammatory status [31,32].

If we consider MS patients, this becomes particularly important when we recognize that long-term disability may develop, leading to a more sedentary lifestyle. While our focus is on the treatment and healthcare of MS patients, we must also prioritize the prevention of cardiovascular diseases and related risk factors, such as a sedentary lifestyle.

Another finding was the significant difference in CK levels between the control group (higher) and the MS group (lower). This could have been due to decreased muscle activity, as physical activity decreases after flare-ups due to weakness, fatigue, medication, or disability from the disease itself [33]. It would be of interest to assess the CK levels in the routine clinical care of these patients, as they are not currently used as a biomarker in the diagnosis or follow-up of MS [34].

In terms of neurotrophic factors, we observed differences in the BDNF levels in the case–control study, where they were higher in the MS group. It is a vital protein for the development, maintenance, and repair of the nervous system, and it also plays an essential role in synaptic plasticity, cognitive function, and mental health. That its values remain elevated after nutritional intervention is of considerable interest, as previous research has shown that BDNF levels in MS have been shown to be decreased [35,36]. Some studies suggest that certain medications, such as Natalizumab, or physical activity may also positively influence BDNF levels [37]. In addition, we found that MS subjects with moderate–intense physical activity after the nutritional intervention had significantly higher BDNF levels. However, those MS patients who engaged in light physical activity had significantly higher levels than the control subjects, with these levels remaining elevated after the nutritional intervention. Mette Diechmann et al. have already described that exercise may contribute to improved serum BDNF levels in people with MS [38]. This may suggest that the most appropriate approach in patients with MS is to add exercise or physical activity in addition to a nutritional intervention, and it would be worth investigating whether exercise in combination with nutrition can stimulate remyelination mechanisms, which is associated with this neurotrophic factor [39]. In contrast to our results, recent studies in patients with relapsing–remitting multiple sclerosis (RRMS) have shown an increase in BDNF levels upon relapse [40].

Another interesting finding is that GDNF levels decreased in MS subjects after nutritional intervention, falling below the baseline levels of the control group. GDNF plays a crucial role in the survival, development, and function of various neurons. However, research on MS has produced mixed results. Some studies have linked elevated GDNF levels to a compensatory glial response to neurodegeneration and inflammation [6]. Similarly, increased expression of GDNF has been observed in areas of brain tissue affected by demyelination in animal models and in post-mortem studies of MS patients [41]. Generally, elevated levels of GDNF may be related to a glial reparative response to neuronal damage, suggesting that increased serum levels could result from a neurorestorative mechanism. As a possible explanation for our findings, we can suggest that the activity of the MIND diet may be related to this decrease in GDNF, because this diet would be able to antagonize inflammation without promoting gliosis.

As is already known, oxidative stress plays a relevant role in the pathogenesis of MS [42,43]. This has been corroborated in the baseline evaluation (case–control study), where we observed a more pro-oxidant profile in the MS group compared with the control group. ROS can induce the degradation of myelin lipids, thereby contributing to demyelination. Additionally, ROS can cause damage to oligodendrocytes. Moreover, ROS are known to activate microglia and macrophages, fostering a pro-inflammatory environment, while also triggering transcription factor activation and directly compromising neuronal axons [44]. However, after 12 weeks of intervention with the MIND diet, we observed a significant decrease in the levels of LPOs and 8-OHdG. LPOs are produced in the presence of a free radical by the oxidation of cellular unsaturated fatty acids in the presence of oxygen. LPO formation results in the destruction of the original lipid, leading to a loss of membrane integrity. Consequently, they can cause a variety of toxic effects in vivo, and their formation is considered a pathological process in biological systems. In MS, it has been proposed that the presence of LPO can reduce the availability of some essential fatty acids and (among other consequences) lead to insufficient incorporation of these fatty acids into cell membranes. Glial cell membrane damage is related to the aetiopathogenesis of MS. This is why LPOs are useful as biomarkers of MS activity [45,46]. 8-OHdG is the common oxidised form of deoxyguanosine in which the C-8 position of the guanine base has a carbonyl group. It can be produced as a consequence of the interaction of ROS with the guanine of DNA. Therefore, 8-OHdG is a useful molecule as a marker of oxidative damage to genetic material and has been postulated to be a sensitive and specific marker of oxidative damage to DNA. As a consequence of this damage, DNA transcription can occur incorrectly, which is associated with tumour formation and progression, cellular ageing and some degenerative diseases (such as MS) [47,48]. It can be said that the MIND diet has antioxidant properties, as it is based on plant-based foods rich in vitamin E (such as nuts, seeds, green leaves, mango, or kiwi), lipoic acid (present in green leafy vegetables, carrots, and potatoes), or resveratrol (present in cocoa, berries, grapes and derived products, almonds, and legumes), and for all these reasons, the MIND diet has contributed to an improvement in the oxidative profile in the MS patients we evaluated. These results would be in line with other research that has related the consumption of diets with known antioxidant effects (such as the Mediterranean Diet) with a decrease in oxidative damage in MS, both in clinical studies and in experimental models [10,20,21].

## 4. Materials and Methods

### 4.1. Study Design

Mixed-design study: cross-sectional study (case–control study) and longitudinal study (before-and-after study) (Figure 1). The longitudinal study was prospective, unblinded, and single-centre. All patients who met the inclusion criteria underwent baseline assessment (T1), and after 12 weeks of nutritional intervention (T2), all tests performed at baseline were performed again.

### 4.2. Study Participants and Ethical Concerns

A total of 44 patients with RRMS were recruited at the Neurology Department of Reina Sofía University Hospital in Córdoba (Spain), from June 2021 to June 2023. Inclusion and exclusion criteria are described in Table 7. In the control group, 40 healthy subjects of similar age and sex to the MS group were recruited. In this group, all those who had any type of disease that interfered with the blood profile determinations were excluded, as well as those who had chronic treatment with drugs with metabolic effects.

Authorization for this study was obtained from the Biomedical Research Ethics Committee of the province of Córdoba. At all times, the harmonized tripartite standards of the Helsinki declaration, the Organic Law on Biomedical Research of 15/1999 of 3 July, the Organic Law on Personal Data Protection (LOPD) of 13 December 2018, the code of ethics of the ‘Organización Médica Colegial’ (OMC), the basic regulatory law 41/2002 on patient autonomy, and rights and obligations regarding clinical information and documentation, as well as the standards of good clinical practice, were respected.

### 4.3. Nutritional Intervention

The diets were designed according to the recommendations and food consumption frequencies outlined by the MIND diet [13], using DietoPro^®^ dietary–nutritional management software (version 27.0).

The nutritional intervention lasted 12 weeks. After this period, a second assessment was conducted, considering the same biochemical and clinical parameters as the initial assessment, to evaluate the effects of the intervention.

To assess adherence to the MIND diet, participants maintained regular telephone and online contact with the professional throughout the intervention period, and adherence was evaluated weekly by the professional. The dietary adherence score was calculated by using the 15 dietary parameters already described [13]. Each parameter was assigned a score of 0, 0.5, or 1, based on its frequency of consumption according to the MIND diet structure. At the end of the intervention, the sum was calculated with a score ranging from 0 to 15 for each participant at each assessment time. A score of <8.5 points was considered low adherence, while a score of ≥9 points was considered high adherence.

### 4.4. Assessment of Parameters

#### 4.4.1. Socio-Demographic Variables and Life Habits

A Registered Dietitian Nutritionist (RDN) conducted individual interviews with each participant to gather information about their lifestyle habits. Lifestyle habits were assessed using a 9-item questionnaire, which collected data on socio-demographic characteristics, sleep duration (< 7 h or ≥ 7 h), and level of physical activity (light–moderate or intense).

#### 4.4.2. Fatigue Scale

The validated Spanish Version of the Daily Fatigue Impact Scale (D-FIS), which assesses fatigue in physical and cognitive terms with 8 items, was used to assess fatigue (typical of MS). This questionnaire was administered to all participants.

#### 4.4.3. Quality of Life in MS

To assess health-related quality of life, the MSQOL-54 scale in Spanish, which is composed of 54 items, was used. The score is assigned according to physical and mental health with values from 0 to 100, with 0 being the lowest (worst quality of life) and the closest to 100 (highest) being the best quality of life. This questionnaire was administered at the beginning and end of the nutritional intervention.

#### 4.4.4. Symbol Digit Modalities Test (SDMT)

For the detection of brain dysfunctions, a neuropsychologist performed the SDMT on the study participants, as it is one of the most sensitive tests proven to check for spontaneous recovery of brain functions and improvements resulting from therapeutic actions [49].

### 4.5. Serum Biomarkers

Fasting blood samples (8 mL) were collected from each participant in tubes with separator gel at each time point of assessment. Samples were centrifuged at 3000 rpm for 5 min, and sera were frozen at −80 °C until analysis.

A routine lab test was performed in which the parameters shown in Table 4 and Table 5 were measured. These determinations were performed on an Inpeco FlexLab^®^ automation chain (Novazzano, Switzerland) with six Siemens Healthcare Diagnostics^®^ ADVIA autoanalysers (Karlsruhe, Germany) mounted on it. Insulin was measured by CMEIA (Chemiluminescent Microparticle Enzyme Immunoassay) on an ADVIA-Centaur XPT module. All other parameters were measured with an ADVIA-Chemistry XPT module by spectrophotometric techniques (enzymatic colorimetric methods). Nfl were determined by SIMoA (Single Molecule Array Technology) by using the Quanterix^®^ (Billerica, MA, USA) single-molecule array enzyme-linked immunoassay HD-X platform. For the determination of neurotrophic factors (BDNF, GDNF, NGF, and GFAP), ELISA kits (Invitrogen^®^, Waltham, MA, USA) were used according to the manufacturer’s instructions.

For the determination of the oxidative stress biomarker 8-OHdG, the New 8-OHdG Check kit (JaICA^®^, Shizuoka, Japan) was used. Lipid peroxidation products (LPOs) and reduced (GSH) and oxidised (GSSG) glutathione were analysed spectrophotometrically with Bioxytech S.A. reagents (Oxis International^®^, Portland, OR, USA) by using a Shimadzu spectrophotometer (UV 1603; Kyoto, Japan).

### 4.6. Statistical Analysis

The Kolmogorov–Smirnov test was performed to check whether the data followed a normal distribution (in which case parametric tests were applied) or a non-normal distribution (in which case non-parametric tests were applied). For the comparison of the means in the case–control study, Student’s *t*-test was used as a parametric test and the Mann–Whitney U-test as a non-parametric test. The Chi-square test was used to compare the categorical variables. For the before-and-after study, a comparison of the two evaluation times was made by using Student’s paired *t*-test (parametric) or Wilcoxon’s *t*-test (non-parametric).

Data are shown as means ± standard deviation ($\bar{x}$ $\bar{X}$ ± SD). If a *p*-value < 0.05, it was considered statistically significant. GraphPad software, Prism 8, version 8.2.1, was used for all statistical analyses.

## 5. Conclusions

This is the first study to investigate a nutritional intervention with the MIND diet in MS patients. Similar to findings in other neurodegenerative diseases, this diet demonstrates neuroprotective effects in MS. The results indicate that this dietary pattern contributes to an improvement in the lipid profile, as well as a decrease in inflammation and oxidative stress. A decrease in the GDNF levels was also observed, which may reflect a reduced need for neuronal repair. Furthermore, adherence to this diet is associated with improvements in patients’ clinical conditions, including reduced fatigue and enhanced physical quality of life.

Therefore, the MIND diet could serve as an adjuvant therapeutic resource for MS patients, in addition to pharmacological, psychological, or physiotherapeutic treatments. This is particularly relevant given that the diet does not involve any toxicity. Additional studies with larger sample sizes, longer intervention periods, and investigations combining dietary interventions with physical activity are needed to further validate these findings.

## Figures and Tables

**Table 1:** Case–control study: socio-demographic characteristics and lifestyle habits.

Parameter	MS Group (n = 44) $\bar{X}$ ± SD	Control Group (n = 40) $\bar{X}$ ± SD	*p*-Value
Age (years)	40.50 ± 10.03	36.82 ± 14.14	0.141
Gender (%)			0.822
Male	32	38	
Female	68	62	
Educational level (%)			0.455
Primary school	13.63	4.54	
High school	45.45	13.63	
University	40.90	72.72	
Place of residence (%)			0.095
Countryside	15.90	5	
City	84.09	95	
Work status (%)			<0.001 *
Unemployed/retired	63.36	20	
Working	36.36	80	
Nighttime sleep hours (%)			0.984
<7 h	52.26	52.50	
≥7 h	47.74	47.50	
Physical activity (%)			0.625
Light	72.72	65	
Moderate–intense	27.27	35	
Smoking (%)			0.044 *
Yes	34.09	15	
No	65.90	85	
Co-habitants (%)			0.729
Alone	6.82	5	
Accompanied	93.18	95	

* *p* < 0.05.

**Table 2:** Case–control-study: biochemical magnitudes.

Parameter	MS Group (n = 44) $\bar{X}$ ± SD	Control Group (n = 40) $\bar{X}$ ± SD	*p*-Value
Glucose (mg/dL)	85.89 ± 8.45	85.1 ± 9.07	0.685
Albumin (g/dL)	4.72 ± 0.24	4.52 ± 0.27	<0.001 *
Total protein (g/dL)	7.09 ± 0.38	7.17 ± 0.34	0.34
Urea (mg/dL)	32.27 ± 6.76	33.1 ± 8.31	0.618
Creatinine (mg/dL)	0.78 ± 0.13	0.81 ± 0.15	0.309
Uric acid (mg/dL)	4.49 ± 1.07	4.77 ± 1.24	0.277
Total cholesterol (mg/dL)	190.3 ± 28.62	182.2 ± 29.28	0.205
HDL (mg/dL)	56.56 ± 14.91	63.56 ± 14.33	0.033 *
LDL (mg/dL)	117 ± 22.7	101.2 ± 23.18	0.003 *
Triglycerides (mg/dL)	85.14 ± 33.7	88.29 ± 38.26	0.693
Bilirubin (mg/dL)	0.69 ± 0.25	0.75 ± 0.39	0.403
LDH (U/L)	188.9 ± 27.39	181.6 ± 34.14	0.288
GGT (U/L)	17.93 ± 11.58	16.33 ± 8.60	0.482
ALT (U/L)	18.91 ± 9.59	23.26 ± 12.45	0.078
CK (mg/dL)	79.5 ± 47.64	151.9 ± 133.7	0.002 *
ALP (U/L)	70.91 ± 19.44	64.16 ± 17.86	0.107
Na (mEq/L)	141.3 ± 2.48	140.7 ± 1.74	0.224
K (mEq/L)	4.29 ± 0.35	4.28 ± 0.33	0.936
Cl (mEq/L)	107.4 ± 3.08	105.1 ± 2.17	<0.001 *
Ca (mg/L)	9.87 ± 0.44	9.65 ± 0.35	0.021 *
P (mg/dL)	3.56 ± 0.51	3.55 ± 0.56	0.953
Iron (mg/dL)	93.42 ± 34.29	92.08 ± 33.44	0.858
Apo AI (mg/dL)	130.2 ± 20.69	145.1 ± 20.14	0.002 *
Apo B-100 (mg/dL)	76.86 ± 17.29	77.5 ± 17.08	0.868
Ratio APO (ApoB/ApoA1)	0.60 ± 0.15	0.54 ± 0.13	0.078
Lp(a) (mg/dL)	31.24 ± 30.82	40.63 ± 36.78	0.215
CRP (mg/dL)	1.43 ± 1.86	1.15 ± 1.03	<0.001 *
HbA1c (%)	5.24 ± 0.25	5.32 ± 0.32	0.247
Insulin (U/L)	7.42 ± 3.04	6.00 ± 2.83	0.035 *
HOMA-IR index	1.64 ± 0.73	1.68 ± 2.96	0.915
Leukocyte count/µL	6.41 ± 2.38	5.72 ± 1.34	0.115
Haemoglobin (g/dL)	14.3 ± 1.27	14.05 ± 1.19	0.364

HDL, high-density lipoprotein; LDL, low-density lipoprotein; LDH, lactate-dehydrogenase; GGT, gamma-glutamyl transferase; ALT, alanine aminotransferase; CK, creatine kinase; ALP, alkaline phosphatase; Na, sodium; K, potassium; Cl, chlorine; Ca, calcium; P, phosphorus; Apo AI, apolipoprotein A-I; Apo B-100, apolipoprotein B-100; Lp(a), lipoprotein a; CRP, C-reactive protein; HbA1c, glycated haemoglobin; HOMA-IR, homeostatic model for insulin resistance. * *p* < 0.05.

**Table 3:** Case–control study: neurofilaments and neurotrophic factors.

Parameter	MS Group (n = 44) $\bar{X}$ ± SD	Control Group (n = 40) $\bar{X}$ ± SD	*p*-Value
Nfl (pg/mL)	12.09 ± 17.84	8.59 ± 5.05	0.235
GDNF (pg/mL)	254.2 ± 436.8	254.2 ± 341.4	>0.999
NGF (pg/mL)	1165 ± 2505	1210 ± 2297	0.933
GFAP (pg/mL)	686.7 ± 235.6	689.5 ± 199.7	0.954
BDNF (ng/mL)	109.3 ± 31.28	59.19 ± 18.10	<0.001 *

Nfl, neurofilaments; GDNF, glial-derived neurotrophic factor; NGF, nerve growth factor; GFAP, glial fibrillary acidic protein; BDNF, brain-derived neurotrophic factor. * *p* < 0.05.

**Table 4:** Before-and-after study: lifestyles and disability degree.

Parameter	Group MS-T1 (n = 44) $\bar{X}$ ± SD	Group MS-T2 (n = 30) $\bar{X}$ ± SD	*p*-Value
EDSS score	3.2 ± 1.82	3.3 ± 2.09	0.686
Work status (%)			0.043 *
Unemployed/retired	63.64	72.42	
Working	36.36	27.58	
Nighttime sleep hours (%)			0.745
<7 h	52.26	50	
≥7 h	47.74	50	
Physical activity (%)			0.801
Light	72.72	60	
Moderate–intense	27.27	40	

* *p* < 0.05. MS-T2 was measured after 12 weeks of nutritional intervention.

**Table 5:** Before-and-after study: biochemical magnitudes.

Parameter	Group MS-T1 (n = 44) $\bar{X}$ ± SD	Group MS-T2 (n = 30) $\bar{X}$ ± SD	*p*-Value
Glucose (mg/dL)	84.27 ± 8.12	85.17 ± 8.00	0.625
Albumin (g/dL)	4.66 ± 0.21	4.57 ± 0.26	0.03 *
Total protein (g/dL)	7.09 ± 0.31	6.98 ± 0.34	0.058
Urea (mg/dL)	32.5 ± 7.03	32.07 ± 6.61	0.712
Creatinine (mg/dL)	0.78 ± 0.12	0.78 ± 0.14	0.766
Uric acid (mg/dL)	4.37 ± 1.21	4.53 ± 1.13	0.155
Total cholesterol (mg/dL)	192.1 ± 27.48	186.9 ± 27.3	0.121
HDL (mg/dL)	58.86 ± 14.93	57.1 ± 15.76	0.350
LDL (mg/dL)	117.8 ± 24.91	112.1 ± 25.49	0.038 *
Triglycerides (mg/dL)	80.85 ± 33.1	72.11 ± 24.16	0.011 *
Bilirubin (mg/dL)	0.68 ± 0.25	0.68 ± 0.31	0.864
LDH (U/L)	190.8 ± 24.07	196.8 ± 41.52	0.381
GGT (U/L)	18.97 ± 13.16	17.53 ± 9.67	0.297
ALT (U/L)	18.77 ± 10.67	17.47 ± 5.96	0.268
CK (mg/dL)	77.13 ± 54.41	80.17 ± 58.10	0.758
ALP (U/L)	70.52 ± 20.94	71.38 ± 22.50	0.640
Na (mEq/L)	140.9 ± 2.56	140 ± 2.32	0.119
K (mEq/L)	4.29 ± 0.39	4.26 ± 0.40	0.630
Cl (mEq/L)	107.4 ± 3.04	106.4 ± 2.88	0.083
Ca (mg/L)	9.83 ± 0.42	9.70 ± 0.48	0.177
P (mg/dL)	3.50 ± 0.48	3.73 ± 0.62	0.064
Iron (mg/dL)	94.83 ± 36.25	79.9 ± 33.14	0.060
Apo AI (mg/dL)	134.1 ± 20.54	133.6 ± 22.16	0.859
Apo B-100 (mg/dL)	78.45 ± 19.39	76.38 ± 13.65	0.485
Ratio APO (ApoB/ApoA1)	0.58 ± 0.15	0.57 ± 0.13	0.702
Lp(a) (mg/dL)	33.04 ± 30.65	36.83 ± 35.54	0.211
CRP (mg/dL)	1.04 ± 1.14	0.66 ± 0.43	0.117
HbA1c (%)	5.29 ± 0.21	5.35 ± 0.21	0.134
Insulin (U/L)	6.79 ± 2.91	6.71 ± 2.99	0.892
HOMA-IR index	1.52 ± 0.75	1.39 ± 0.59	0.128
Leucocyte count/µL	6.57 ± 2.38	6.61 ± 2.22	0.886
Haemoglobin (g/dL)	14.04 ± 1.32	13.88 ± 1.40	0.405

HDL, high-density lipoprotein; LDL, low-density lipoprotein; LDH, lactate-dehydrogenase; GGT, gamma-glutamyl transferase; ALT, alanine aminotransferase; CK, creatine kinase; ALP, alkaline phosphatase; Na, sodium; K, potassium; Cl, chlorine; Ca, calcium; P, phosphorus; Apo AI, apolipoprotein A-I; Apo B-100, apolipoprotein B-100; Lp(a), lipoprotein a; CRP, C-reactive protein; HbA1c, glycated haemoglobin; HOMA-IR, homeostatic model for insulin resistance. * *p* < 0.05.

**Table 6:** Before-and-after study: neurofilaments and neurotrophic factors.

Parameters	Group MS-T1 (n = 44) $\bar{X}$ ± SD	Group MS-T2 (n = 30) $\bar{X}$ ± SD	*p*-Value
Nfl (pg/mL)	14.11 ± 21.26	19.53 ± 26.78	0.282
GDNF (pg/mL)	240.5 ± 441.9	198.8 ± 274.9	0.041 *
NGF (pg/mL)	1012 ± 2269	855.7 ± 1943	0.122
GFAP (pg/mL)	697.2 ± 257.6	676.9 ± 241	0.495
BDNF (ng/mL)	106.8 ± 34.54	109.9 ± 32.14	0.603

Nfl, neurofilaments; GDNF, glial-derived neurotrophic factor; NGF, nerve growth factor; GFAP, glial fibrillary acidic protein; BDNF, brain-derived neurotrophic factor. * *p* < 0.05.

**Table 7:** Inclusion and exclusion criteria.

Inclusion Criteria	Exclusion Criteria
Be diagnosed with MS according to McDonald’s criteria (2017); Have a score from 0 to 6.5 on the EDSS scale; >18 years old; Under treatment with natalizumab, alemtuzumab, ocrelizumab, rituximab, or cladribine; Be able to understand the purpose of the study; Be able to perform all procedures required by the study protocol; Give signed informed consent; Having been previously evaluated by a neurologist of the department.	Being in a disease outbreak or presenting a clinical situation that indicates that the disease is not stabilised or controlled; Be admitted to hospital for any other reason (intercurrent disease or surgical intervention); Changes in medication for at least the last two months; Being pregnant or breastfeeding.
